# AAV-Mediated Administration of Myostatin Pro-Peptide Mutant in Adult *Ldlr* Null Mice Reduces Diet-Induced Hepatosteatosis and Arteriosclerosis

**DOI:** 10.1371/journal.pone.0071017

**Published:** 2013-08-01

**Authors:** Wen Guo, Siu Wong, Shalender Bhasin

**Affiliations:** 1 Department of Medicine, Boston University School of Medicine, Boston, Massachusetts, United States of America; 2 Research Program in Men’s Health: Aging and Metabolism, Boston Claude D. Pepper Older Americans Independence Center for Function Promoting Anabolic Therapies, Brigham and Women’s Hospital, Harvard Medical School, Boston, Massachusetts, United States of America; University College London, United Kingdom

## Abstract

Genetic disruption of myostatin or its related signaling is known to cause strong protection against diet-induced metabolic disorders. The translational value of these prior findings, however, is dependent on whether such metabolically favorable phenotype can be reproduced when myostatin blockade begins at an adult age. Here, we reported that AAV-mediated delivery of a myostatin pro-peptide D76A mutant in *adult mice* attenuates the development of hepatic steatosis and arteriosclerosis, two common diet-induced metabolic diseases. A single dose of AAV-D76A in adult *Ldlr* null mice resulted in sustained expression of myostatin pro-peptide in the liver. Compared to vehicle-treated mice, D76A-treated mice gained similar amount of lean and fat mass when fed a high fat diet. However, D76A-treated mice displayed significantly reduced aortic lesions and liver fat, in association with a reduction in hepatic expression of lipogenic genes and improvement in liver insulin sensitivity. This suggests that muscle and fat may not be the primary targets of treatment under our experimental condition. In support to this argument, we show that myostatin directly up-regulated lipogenic genes and increased fat accumulation in cultured liver cells. We also show that both myostatin and its receptor were abundantly expressed in mouse aorta. Cultured aortic endothelial cells responded to myostatin with a reduction in eNOS phosphorylation and an increase in ICAM-1 and VCAM-1 expression. Conclusions: AAV-mediated expression of myostatin pro-peptide D76A mutant in adult *Ldlr* null mice sustained metabolic protection without remarkable impacts on body lean and fat mass. Further investigations are needed to determine whether direct impact of myostatin on liver and aortic endothelium may contribute to the related metabolic phenotypes.

## Introduction

Metabolic disorders, such as diabetes mellitus, nonalcoholic fatty liver disease, and arteriosclerosis are leading causes of morbidity and mortality in modern world. With the aging of human populations, the prevalence of these inter-linked metabolic disorders is increasing globally. We show here that myostatin, a muscle-secreted growth and differentiation factor, may be a potential therapeutic target for the prevention and treatment of these metabolic disorders. Genetic disruption of myostatin gene causes marked hypermuscularity and hypoadiposity [1]–[4]. Genetic inactivation of myostatin in *Ldlr* null mice also alleviates diet-induced hepatosteatosis and arteriosclerosis [5]. However, it is not known whether similar metabolic improvement can be achieved by myostatin antagonists in adults, an issue that is crucially important for the relevant clinical applications.

We show here that administration of a protease-resistant myostatin pro-peptide D76A mutant to adult mice, using the AAV technology, induced a substantial reduction in liver fat infiltration and aortic atheromatous lesions with only a mild impact on body fat and lean mass. The strong association between this metabolically favorable phenotype and robust ectopic expression of myostatin pro-peptide in the liver of the D76A-treated mice, with minor changes in muscle and fat mass, led us to consider the hypothesis that myostatin may have direct effects on hepatic lipid metabolism. We show here for the first time that myostatin increases *de novo* hepatic lipogenesis in cultured liver cells. In addition, we show that myostatin and its receptor are both abundantly expressed in mouse aorta. Exposure of aortic endothelial cells to myostatin resulted in activation of TGFβ signaling and reduced phosphorylation of endothelial NO synthase (eNOS) in association with increased expression of pro-atherogenic adhesion molecules ICAM-1 and VCAM-1. Our results indicate that both liver and endothelium are direct targets of myostatin which may be involved in diet-induced metabolic disorders.

## Materials and Methods

### Materials

Myostatin pro-peptide D76A mutant fused with mouse IgG-Fc was a gift from Dr. Se-Jin Lee (Johns Hopkins University, Baltimore, MD [6]). The recombinant product of this construct (D76A-Fc) has been shown to moderately increase skeletal muscle mass in adult mice [6], [7]. The construct was used to generate an adeno-associated virus (AAV9), using a commercial service (Vector Biolabs, Philadelphia, PA), for sustained ectopic expression of the myostatin pro-peptide D76A mutant [7]–[9]. Recombinant myostatin mature peptide of mammalian origin was provided by Amgen Inc (Thousand Oaks, CA). Luciferase reporter for TGFβ/myostatin signaling (3TP-Lux) was obtained from Addgene (#11767, [10]). Renilla luciferase vector (#E6891) and dual luciferase reporter assay kit (#E1910) were from Promega (Madison, WI).

### Animals and Diet

The animal protocol was approved by IACUC of Boston University. Male *Ldlr* null mice were purchased from Jackson Laboratory (Bar Harbor, MI). At eight week of age, mice were analyzed by NMR and divided into two groups of similar body composition. Animals were injected through tail vein with either vehicle (saline) or AAV-D76A (4×10^11^ vg/ea, diluted in saline), a dose similar to those used in previous studies [7]. AAV with no functional gene products does not cause phenotypic changes in *Ldlr* ^−/−^ mice [11]. This was confirmed in our preliminary studies with AAV-eGFP (not shown). Animals were maintained on regular chow diet for 7 weeks to stabilize post-injection body composition. All mice were then switched to a high fat diet (TD.09547, Harlan Laboratories, Madison WI) for 12 weeks. This diet contains 48.4%, 21.4%, and 30.1% calories from carbohydrate, protein, and fat, respectively, as well as 0.05% cholesterol.

### Aortic Lesion Identification

After euthanasia, the aorta was stained *en face* with Sudan IV for lipid-rich lesions as described before [5], [12]. Degree of atherosclerosis in the exposed aorta was presented as the percentage of lesion-covered area in the tissue surface using NIH Image J program. Sections of the aortic root (5 µm) near the sinus were stained with hematoxylin and eosin (H&E) and immuno-stained against MAC-3 and VCAM-1, as described [5], [12]. Results of immuno-staining was scored blindly following a modified score system as described before (http://www.ihcworld.com/ihc_scoring.htm). Masson’s Trichrome staining was performed using commercial reagents (Market Lab Inc. Caledonia, MI).

### Other Tissue Analysis

Selected fat depots, muscle groups, and liver were weighed and expressed as a percentage of total body weight. Liver lipid staining and tissue analyses for protein and RNA expression were performed as described [5], [12]. Expression of D76A in liver and muscle was measured using real-time PCR using primers designed for myostatin pro-peptide (NM_010834, nt 222–414, which encompasses the D76A mutation site). For the liver, the endogenous myostatin expression was low. Administration of AAV-D76A induced a large increase in the expression level of pro-peptide mRNA (Figure S1), a clear demonstration of robust expression of the ectopic gene. In contrast, we were not able to detect significant increase in muscle expression of myostatin pro-peptide (Figure S1, right panel) or mature peptide (primer set for nt 1015–1145, NM_010834, data not shown). Because full-length myostatin (a single gene that encodes both pro-peptide and mature peptide) is strongly expressed in muscle, the lack of difference in pro-peptide expression between vehicle and D76A-treated mice indicates that either the ectopic gene was not expressed or its expression was low enough not to cause a detectable addition to the endogenous gene. These results are consistent with previous reports that AAV9, when injected through the tail vein, is primarily expressed in the liver [9], [13], [14].

### Plasma Lipids

Fasting blood samples (no food between 10 pm to 10 am) were collected 10 weeks after high fat diet. Plasma lipids were measured as described previously [5], [12].

### Insulin Tolerance and Pyruvate Tolerance

These tests were performed during week 11 after introduction of the high fat diet. Briefly, insulin (Humalog, USP**)** was diluted in sterile saline containing 0.1% BSA and injected (0.6 U/kg, 0.1 ml, i.p.) after 4 h fasting. Sodium pyruvate (Gibco #11360-070) was diluted in saline and injected (1.5 g/kg, 0.1 ml, i.p.) after overnight fasting. Blood glucose was measured at baseline and every 15 min after the injection for 2 hours.

### Cell Culture

Human hepatoma HepG2 cells (a gift from Dr. Mengwei Zang, Boston University) were grown as described [15]. Cells were switched to low glucose DMEM (Gibco # 11885084) containing 1% fetal bovine serum (Gibco # 10438-018) overnight and then treated with myostatin for different incubation periods and harvested for Western or RT-PCR Analysis. Bovine aortic endothelial cells (a gift from Dr. Yasuo Ido, Boston University) were grown as described [16] in low glucose DMEM supplemented with 10% calf serum (Sigma-Aldrich, #12133C) and primary human aortic endothelial cells (Invitrogen, #C-006-5C) were grown in a vendor-specified medium (Invitrogen, #C00625PA). Cells were switched to serum-free medium overnight before treatments.

### Assays for Fatty Acid Oxidation and De Novo Fatty Acid Synthesis

HepG2 cells were pre-labeled with trace amount of [9,10-^3^H] oleate overnight (2 µCi per well in a 6 well plate), washed to remove unincorporated tracers and incubated with myostatin (100 ng/ml) for 24 h. Cells were washed again and incubated with serum-free medium containing the same concentrations of myostatin for another 8 hours. Acid-soluble metabolites released from the cells were counted as the index for fatty acid oxidation [17]. Similarly, HepG2 cells were incubated with myostatin for 24 hours and [1,2-^14^C] acetic acid was added for the last 3 hours of the incubation. Lipid extracts were separated by TLC (Hexane : ether : acetic acid at a ratio of 70∶30:1) and visualized under iodine vapor. The triglyceride-incorporated radioactivity was counted as an index of fatty acid synthesis. Total cellular triglyceride content was measured after 48 hour incubation and normalized to cellular protein.

### Immunohistochemistry

Mouse aorta root was fixed in formalin and sections were prepared as described before [12]. Immunohistochemistry was performed using a Zymed HistoMouse-SP Kit (AEC, Broad Spectrum, Invitrogen #959544), following the manufacturer’s instructions. Antibody for MAC3 was purchased from BD Biosciences (#550292; San Jose, CA). Antibody for VCAM-1 was from Santa Cruz (#SC-1504, Santa Cruz, CA).

### Western Analysis

Tissue and cellular homogenates were prepared for Western analysis as described before [12]. First antibodies were purchased from Cell Signaling (eNOS, #9586; phosphor-eNOS-Ser1177, #9570; phosphor-Smad3, #9520) and Santa Cruz (VCAM-1, #SC-1504; beta-tubulin, #SC-9104; ActRIIB, #SC-5665; and Smad2/3, #SC-6032).

### Statistical Analysis

All numerical results are presented as means±SEM. Comparison between two treatments were performed using Student’s *t* test. Comparisons among multiple groups were performed using ANOVA. Unless specified, all results obtained in tissue culture experiments are representative of at least three experiments and results obtained in tissue samples are representative of 5–10 animals of each group.

## Results

### Effects of D76A on Body Composition

Consistent with previous reports [7], we observed a modest increase in lean body mass in mice following the injection of AAV-D76A while the mice were maintained on a low fat chow (Figure S2A). Seven weeks after the injection of AAV-D76A, the animals were switched to a high fat diet to study the effect of D76A on diet-induced metabolic responses. During the period of high fat diet, the changes in lean and fat mass were similar between the vehicle and D76A-treated animals (Figure S2B). Mice were euthanized after twelve weeks of high fat diet, and individual tissues were weighed. D76A-treated mice showed a 15% increase in quadriceps and 5–10% increase in gastrocnemius and levator muscle groups compared to the control group (Figure S2C). These differences were relatively small as compared with the 100–150% increase reported in the myostatin null mice [1]–[5] and were likely acquired during the initial low fat feeding period. D76A-treated mice showed a small decrease in inguinal fat but fat mass in perirenal, epididymal, and mesenteric depots were not different between the two groups (Figure S2C). This is in striking contrast to the global hypoadiposity found in mice with life-long genetic myostatin blockade [1]–[5], [18], but in line with a recent study on diet-induced metabolic disorders in mice with adult age myostatin deletion [19].

### Effects of D76A on Aortic Atherosclerosis

**Figure 1:**
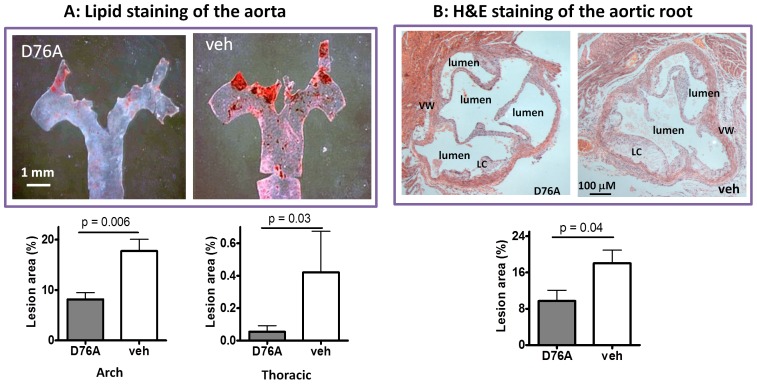
AAV-mediated expression of myostatin pro-peptide D76A mutant attenuates aortic atheromatous lesion accumulation in *Ldlr* null mice. The animals were injected with either AAV-D76A (D76A) or vehicle (veh) at 8 weeks of age, placed on a high fat diet seven weeks later, and euthanized after another 12 weeks. The entire aorta along with the aortic root was dissected for analysis. (**A**) *En face* staining with Sudan IV for lipid-rich lesions (stained red). The lower panel shows the quantitative lesion scores (mean ± SEM, n = 10). (**B**) Sections of the aortic root near the sinus stained with hematoxylin and eosin (H&E) for lesions built up along the vessel wall (LC: lipid core, VW: vessel wall). The lower panel shows the lesion area expressed as a percent of aortic area (mean±SEM, n = 10).

The atheromatous lesions in the aorta were analyzed using the *en face* method [5], [12]. Most of the lesions were found in the aortic arch and fewer lesions were present in the thoracic and abdominal regions. D76A-treated mice had significantly fewer lesions than vehicle-treated mice (Figure 1A, additional images of aorta lesion staining are displayed in Figure S3). The mean lesion scores in the aortic arch and the thoracic aorta were 57% and 83% lower in D76A-treated animals than those in the control mice, respectively (Figure 1A, lower panel). Hematoxylin and eosin staining of the sections near the sinus of the aortic root revealed a significant reduction of lesion size in D76A-treated mice compared with the control mice (Figure 1B), consistent with the *en face* assessment. Similar results were observed by trichrome staining of the aortic roots, which further revealed a decrease in lipid core size and the cholesterol clefts in mice treated with D76A (Figure S4).

**Figure 2:**
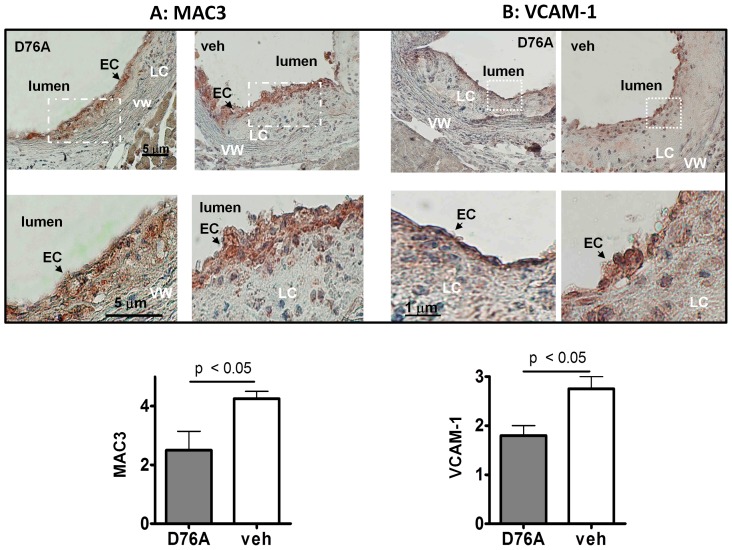
AAV-D76A reduces expression of MAC-3 and VCAM-1 in aortic lesions. Sections adjacent to the sinus of the aortic roots were immuno-stained using antibodies against (**A**) CD107b (MAC-3), a marker of macrophage infiltration, and (**B**) vascular cell adhesion molecule (VCAM-1), an endothelial ligand that promotes the adhesion of lymphocytes, monocytes, eosinophils, and basophils (EC: endothelial cells, VW: vessel wall, LC: lipid core). Lower panels display the quantitative analyses of immunostaining determined by densitometry (mean±SEM, n = 5).

To assess the inflammatory status of the lesions, we performed immunostaining of the aortic root sections for MAC3, a marker of macrophage infiltration. As shown in Figure 2A, vehicle-treated mice showed greater MAC3 staining than D76A-treated mice. At higher magnification, the lesions from D76A-treated mice were found to contain fewer and less intensely stained MAC3-positive cells than those from vehicle-treated mice (Figure 2A). Densitometric analysis revealed 41% reduction in MAC3 staining in AAV-D76A-treated mice compared to controls (Figure 2A, lower panel). As endothelial inflammation usually begins with the adhesion of circulating inflammatory cells, an event facilitated by the vessel wall production of *vascular cell adhesion molecule-1* (VCAM-1), we also stained the aortic root sections for VCAM-1. As shown in Figure 2B, D76A treatment reduced VCAM-1 staining by 30% compared to vehicle treatment.

### Effects of D76A on Plasma Lipid Profile and Insulin Sensitivity

**Figure 3:**
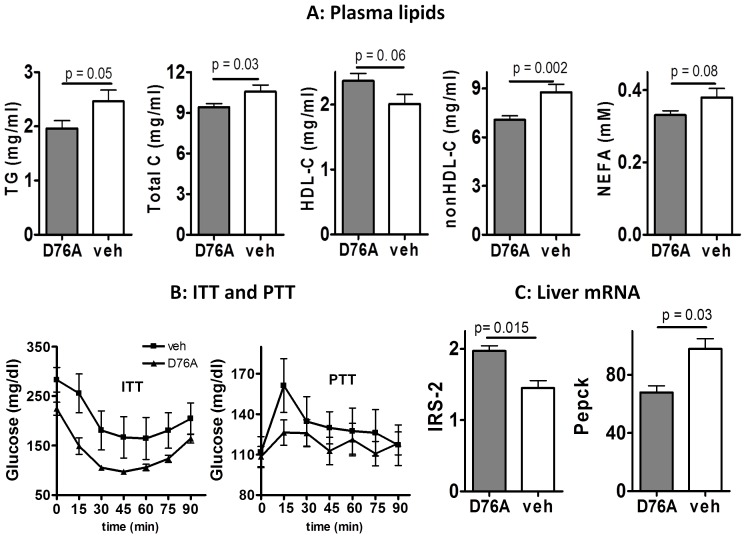
AAV-D76A regulates plasma lipid profile and insulin sensitivity. (**A**) Plasma lipid profile: fasting plasma samples were obtained after 10 weeks of high fat feeding and analyzed for triglycerides (TG), total cholesterol (Total C), HDL cholesterol (HDL-C), non HDL cholesterol (nonHDL-C), and non-esterified fatty acids (NEFA). (**B**) Insulin tolerance test (ITT) and pyruvate tolerance test (PTT) performed after 11 weeks of high fat feeding. For ITT, food was removed in the morning for four hours and then animals were injected with an insulin bolus (0.6 U/kg diluted in sterile saline containing 0.1% albumin, i.p.). For PTT, food was removed overnight for 16 hours and then animals were injected sodium pyruvate (1.5 g/kg, i.p. diluted in sterile saline). Blood glucose was measured using a glucometer and was plotted against time before and after the injections (n = 10, mean ± SEM). (**C**) Liver mRNA expression of insulin receptor substrate-2 (IRS-2) and phosphoenolpyruvate carboxykinase (Pepck) was analyzed by real-time PCR (mean±SEM, n = 10).

Plasma cholesterol circulates in different forms of lipoproteins, of which HDL is generally anti-atherogenic and the non-HDL lipoproteins with variable triglyceride to cholesterol ratios are proatherogenic. A high ratio between non-HDL and HDL cholesterol usually correlates with accelerated lesion development, and vice versus. As shown in Figure 3A, D76A treatment was associated with lower plasma levels of triglycerides (p = 0.05), total cholesterol (p = 0.03), and non-HDL cholesterol (p = 0.002), and a trend towards higher HDL cholesterol (p = 0.06) and lower free fatty acids (p = 0.08), in comparison to vehicle treatment. The difference in each measurement, however, is relatively small as compared to that observed in mice with genetic myostatin knockout [5], considering that D76A-treatment correlates with a greater suppression of aortic lesions. Whether D76A specifically regulates subclass of pro-atherogenic plasma components, such as oxidized lipoproteins, requires for further investigations.

**Figure 4:**
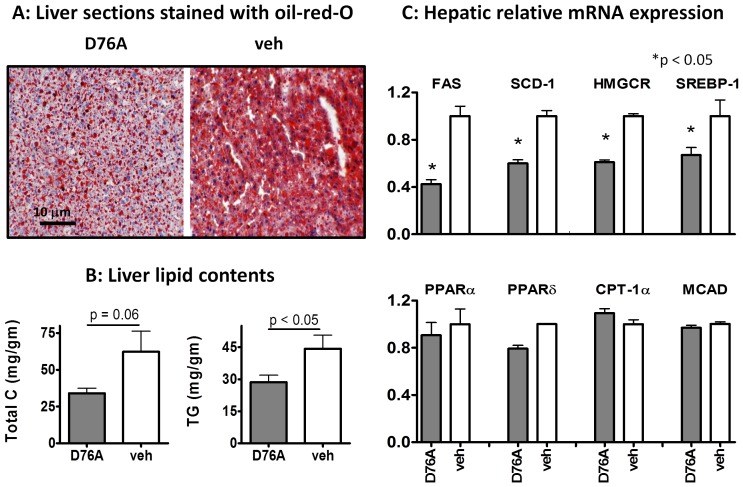
AAV-D76A attenuates liver fat infiltration, reduces liver triglycerides and cholesterol, and down-regulates mRNA expression of selected lipogenic genes. (**A**) Cryo-sectioned liver samples (10 m) were stained with oil-red-O (red for lipids). Results are representative of three animals per group. (**B**) Liver lipids were extracted by Folch’s method and analyzed for total cholesterol (Total C) and triglycerides (TG) using commercial kits (mean ± SEM, n = 10). (**C**) Liver mRNA expression was analyzed using qPCR; the upper panel shows genes involved in lipid synthesis and the lower panel shows the genes involved in lipid oxidation (FAS: fatty acid synthase, SCD-1: stearoyl-CoA desaturase-1, HMGCR: 3-hydroxy-3-methyl-glutaryl-CoA reductase, SREBP-1c: sterol regulatory element-binding protein 1, PPAR: peroxisome proliferator-activated receptor, CPT: carnitine palmitoyl acyltransferase, MACD: medium chain acylCoA dehydrogenase). All PCR results were normalized to the expression level of the house-keeping gene HPRT (mean ± SEM, n = 10).

Since a proatherogenic plasma lipid profile and insulin resistance are often associated [20], we investigated the effects of D76A treatment on insulin sensitivity. Fasting glucose (111±12 vs. 107±13 mg/dl, respectively) and fasting insulin concentrations (2.95±0.7 vs. 2.8±0.5 ng/ml, respectively) were similar between the two groups. However, in the non-fasting state (4 h after food removal in the morning), plasma glucose concentration was lower in D76A-treated mice than in the controls (Figure 4B, left panel). Since mice were provided free food access except for occasional fasting (two days out of 3 months), non-fasting results would be more physiologically relevant, which suggests that D76A-treated animals have greater steady-state insulin sensitivity than the control animals.

In response to a bolus injection of insulin, the kinetic change of blood glucose did not differ between the two groups, but the plasma glucose concentrations decreased to a lower level in the D76A-treated mice than in the control mice. The area-under-the-glucose-curve was significantly lower in D76A-treated mice than in the vehicle-treated mice (AUC glucose 11629 vs. 17880 mg×min/dL for D76A- and vehicle-treated mice, respectively, p = 0.006). These results suggest that D76A-treated mice had better glycemic control under steady-state condition but their acute response to insulin administration was not dissimilar from that in vehicle-treated animals. Because insulin-stimulated glucose uptake occurs primarily in skeletal muscle, these results suggest that treatment with D76A likely did not have a major impact on skeletal muscle insulin sensitivity. Western analysis for selected key elements in the insulin signaling cascade, including phosphorylated Akt and p70S6, in the skeletal muscle and the adipose tissue revealed no significant difference between the two groups (not shown). These results contrast the findings in mice with genetic myostatin blockade for which skeletal muscle is hyper-sensitive to acute insulin administration [1]–[3], [5], [18] and highlights the phenotypic difference between life-long genetic myostatin blockade and adult age treatment with myostatin antagonists.

Unlike muscle and fat that take up glucose in response to insulin, liver produces glucose in response to nutrient deprivation, a process that is potently inhibited by insulin [21]. An increase in basal (non-fasting) plasma glucose concentration can be a sign of impaired insulin sensitivity in the liver so that it continues to produce glucose even when nutrient is plentiful (Figure 4B, left panel). Hence, we performed the pyruvate tolerance test, which measures the rise of blood glucose in response to a bolus injection of pyruvate. As shown in Figure 3B (right panel), blood glucose concentrations rose faster and to a higher level in the control mice than in D76A-treated mice, suggesting that the latter had a greater restraint on hepatic glucose output. Real-time PCR analysis showed that D76A-treated mice had higher hepatic expression levels of insulin receptor substrate 2 (IRS-2, Figure 3C) but not IRS-1 (not shown). Of note, IRS-2 has been reported to play a more important role than IRS-1 in hepatic insulin action [22]. Consistently, D76A-treated animals also displayed lower liver expression of Pepck (Figure 3C), a key enzyme for gluconeogenesis known to be transcriptionally suppressed by insulin [5], [23]. Together, these results suggest that D76A-treated animals have improved hepatic insulin sensitivity in comparison to vehicle-treated mice under our experimental conditions.

### Effects of D76A on Hepatosteatosis and Hepatic Metabolic Gene Expression

Fatty liver is linked to diet-induced dyslipidemia and atherosclerosis [24]. We found that D76A-treated mice had significantly lower liver fat than the control mice, as indicated by lipid staining of liver sections (Figure 4A) and by lipid analysis of the liver extracts (Figure 4B). D76A-treated mice also had lower hepatic mRNA expression of key enzymes involved in lipogenesis, including fatty acid synthase (FAS), stearoyl CoA dehydrogenase (SCD-1), and HMG CoA reductase (HMGCR) (Figure 4C, upper panel). These enzymes are largely regulated at the transcriptional level [25]. Consistently, we found that liver expression level of SREBP-1c, the master transcription regulator of hepatic lipogenic enzymes [26], [27], was lower in D76A-treated animals than in the controls. In contrast to its inhibitory effect on lipogenic gene expression, D76A treatment had no effect on hepatic expression of important genes involved in fatty acid oxidation (Figure 4C, lower panel). D76A treatment also did not affect the expression of selected inflammatory cytokines, such as TNFα, MCP-1, and CRP (Figure S4). Plasma IL-6 and MCP-1 were very low and were not significantly affected by D76A (data not shown). The expression levels of selected stress markers, C/EBP homologous protein-10 (CHOP-10) and heme oxygenase 1 (HO-1), were higher in the liver of control mice than D76A-treated mice (Figure S5), consistent with the notion that fat infiltration induces expression of stress genes [28].

### Liver is a Novel Direct Target of Myostatin

**Figure 5:**
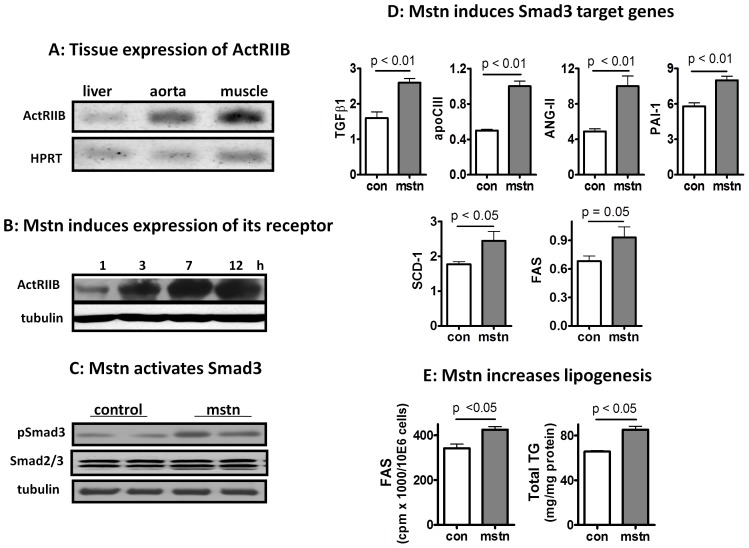
Liver is a direct target of myostatin signaling. (**A**) PCR measurement of the expression of myostatin receptor, ActRIIB, in the liver, aorta, and skeletal muscle. (**B**) Myostatin induces sustained expression of its own receptor in HepG2 cells (mstn, 100 ng/ml, harvested at indicated time points). (**C**) Myostatin induces Smad3 phosphorylation in HepG2 cells (100 ng/ml, 30 min). All results shown in (A–C) are representative of more than three independent measurements. (**D**) Myostatin (100 ng/ml, 24 h) induces expression of Smad3 target genes in HepG2 cells (mean ± SEM, n = 4). (**E**) Myostatin (100 ng/ml) induces lipogenesis and lipid accumulation in HepG2 cells (means ± SEM, n = 4).

Because the atheroprotective phenotype mediated by AAV-D76A injection was only weakly associated with changes in muscle mass, but strongly associated with changes in hepatic fat and robust hepatic expression of ectopic D76A, we considered the possibility that metabolic protection may be a result of direct blockade of myostatin signaling in the liver. To our knowledge, direct effect of myostatin on liver has not been reported although several studies have speculated such a possibility [3], [29], [30]. There has been some uncertainty whether ActRIIB, the high affinity receptor for myostatin [31], [32], is expressed in the liver [33]–[36]. Using RT-PCR, we confirmed that ActRIIB was expressed in the mouse liver although to a moderate extent (Figure 5A). When recombinant myostatin was added to liver cells (HepG2) in culture, ActRIIB expression was induced in a sustained manner (Figure 5B). Similar induction of ActRIIB was confirmed in isolated primary mouse hepatocytes (Figure S6). Myostatin rapidly induced Smad3 phosphoylation (Figure 5C), an essential component in the canonical myostatin signaling cascade [37], [38]. Myostatin also increased the expression of selected genes downstream of Smad3; including TGFβ1, apoCIII, PAI-1, and angiotensinogen II (AngII) (Figure 5D). PAI-1 and apoCIII are known to be transcriptionally regulated by Smad3 [39], [40], whereas AngII can be induced by TGFβ1 via a Smad-independent pathway [41]. In line with these in vitro findings, we show that inhibition of myostatin by D76A was associated with decreased expression of TGFβ1 and apoCIII in the liver (Figure S5, lower panel). In addition, cells treated with myostatin also showed increased expression of enzymes involved in lipogenesis, such as SCD-1 and FAS (Figure 5D, lower panel). Consistent with the increase in the expression of lipogenic genes, we found that myostatin increased fatty acid synthesis and lipid accumulation in cultured liver cells (Figure 5E). There was no effect of myostatin on fatty acid oxidation measured in parallel (data not shown). To our knowledge, this is the first report of direct pro-lipogenic effect of myostatin on liver cells, although others have shown that TGFβ1, which shares similar post-receptor signaling pathways with myostatin, increases SCD-1 expression and increases lipid storage in cultured liver cells [42], [43].

### Aortic Endothelium can be another Direct Target of Myostatin

**Figure 6:**
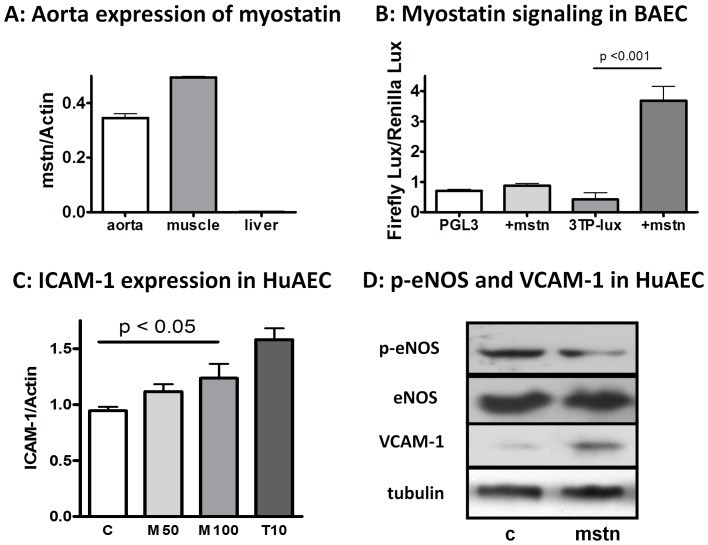
Aorta is a direct target of myostatin signaling. (**A**) PCR analysis of aortic expression of endogenous myostain mature peptide (mstn, nt 1015–1145, NM_010834), using skeletal muscle as the positive control and liver as the negative control (mean ± SEM, n = 3). Of note, while actin is often considered as an abundant house-keeping gene in multiple tissues, its expression level may not be cross-comparable. Hence, the results presented here only demonstrated that myostatin is abundantly expressed in the aorta but does not indicate that the expression is to a similar level found in the muscle. (**B**) Myostatin induces activation of its reporter gene 3TP-Lux in bovine aortic endothelial cells (BAEC, mean ± SEM, n = 3). (**C**) Myostain induces ICAM-1 expression in primary cultured human aortic endothelial cells (HuAEC). TGFβ1 (T: 10 ng/ml) served as a positive control (M50: myostatin 50 ng/ml, M100∶100 ng/ml, 3 h incubation in serum-free Medium 199 supplemented with oxLDL 0.025 mg/ml to raise the basal expression of ICAM-1) (mean ± SEM, n = 4). (**D**) Myostatin (100 ng/ml) down regulates the expression of phosphor-eNOS and up regulates VCAM-1 expression in HuAEC. Results are reflective of three independent experiments.

As shown in Figure 5A, the high affinity myostatin receptor, ActRIIB, was robustly expressed in mouse aorta, so was endogenous myostatin gene (Figure 6A). Within the aortic endothelium, endothelial cells represent the first line of defense against the insult from circulating factors. We thus evaluated the direct effects of myostatin on aortic endothelial cells. Similar to that found in the liver cells (Figure 5C), myostatin induced robust Smad3 phosphorylation in both bovine and primary human aortic cells (not shown). Figure 6B demonstrated that myostatin directly induced activation of 3TP-Lux reporter gene for TGFβ signaling in bovine aortic endothelial cells, an effect that requires cooperation between both type-II and type-I receptors [10]. Since type-I receptors are shared between myostatin and TGFβ [44], the induction of 3TP-Lux by myostatin confirms functional activation of myostatin signaling in the aortic endothelial cells. Furthermore, incubation with myostatin induced the expression of intracellular cell adhesion molecule 1 (ICAM-1, Figure 6C), down-regulated steady-state phosphorylation/activation of endothelial NO synthase (eNOS, Figure 6D) and up-regulated the expression of vascular cell adhesion molecule (VCAM-1, Figure 6D) in primary cultured human aortic endothelial cells (HuAEC). As eNOS protects the endothelium against insults from circulating inflammatory cytokines and oxidized lipoproteins [45] while ICAM-1 and VCAM-1 promote monocyte adhesion and lesion development [46], these results suggest that myostatin blockade may have a favorable effect on the endothelium under certain physiological conditions, such as hyperlipidemia, by reducing local expression of pro-adhesion molecules, a hypothesis that requires further investigations.

## Discussion

Our data provide the first evidence that AAV-mediated administration of myostatin pro-peptide D76A mutant in adult mice attenuates diet-induced aortic atherosclerosis and hepatosteatosis in *Ldlr* null mice. These data provide a new rationale to support the development of anti-myostatin strategies for the prevention and treatment of atherosclerosis and hepatosteatosis in humans.

It is important to emphasize that although genetic modulation is useful to validate a concept, the feasibility for translational medicine is entirely dependent on whether a favorable phenotype of a genetic model can be successfully recapitulated by post-natal, especially adult age, interventions. Life-long genetic myostatin blockade causes dramatic hyper-muscularity and hypo-adiposity [1]–[3], [5]. Hence, it is not clear whether the associated metabolic phenotype, i.e. strong protection against diet-induced insulin resistance, hepatosteatosis, and atherosclerosis [1]–[3], [5], is directly related to myostatin signaling or secondary to the extreme muscle hypertrophy. Indeed, it has been shown that life-long muscle-specific transgenic myostatin blockade was sufficient to reproduce the phenotype of mice with global myostatin knockout, unequivocally proving that dramatic muscle hypertrophy alone is sufficient to cause global metabolic protections [1], [3], [18]. However, such extremely hypertrophic muscle phenotype was not reproduced in mice with adult age myostatin inhibition [6]–[9], [19], [47](this work). In addition, life-long genetic myostatin blockade, global or muscle-specific, prevents diet-induced increment in fat mass [1]–[3], [5], a phenotype that was also not reproducible in mice with adult age myostatin inhibition ([19], this work). However, despite a modest impact on body composition, adult age myostatin inhibition does prove effective in protection against diet-induced fatty liver ([19], this work) and atherosclerosis (this work), thus prompting us to consider organ-specific effect of myostatin (and its inhibitors) on metabolic regulations without being overwhelmed by the hyper-muscular phenotype found in mice with life-long genetic myostatin blockade.

Although protection against diet-induced hepatosteatosis has been documented in mice with genetic myostatin blockade for life or induced at adult age [1]–[3], [5], [19], it is generally considered as secondary to the impact on muscle health [1], [30]. To our knowledge, the results provided in this work are the first evidence that myostatin can participate in hepatic metabolic regulation through direct interaction with liver cells. We show that liver cells express high affinity receptor for myostatin and respond to myostatin stimulation by increasing phosphorylation of Smad3 and expression of Smad3-responsive genes including SCD-1 [42], a key lipogenic gene in fatty liver development [48]. Furthermore, we have shown that myostatin induces the expression of selected lipogenic genes and increases lipid accumulation in the cultured liver cells. This finding is congruent with the reports that TGFβ1, which shares the type I receptor with myostatin, induces lipogenic gene expression and increases lipid accumulation in liver cells [42], [43].

A second novel finding reported in this work is the direct effect of myostatin on aortic endothelial cells. While liver plays a central role in systemic lipid metabolism, aortic atherosclerosis starts with endothelial damage and monocyte adherence, which set the stage for lipid uptake and foam cell formation. We show here that myostatin and its receptor are robustly expressed in the aortic tissue. In addition, we provide evidence that myostatin directly interacts with cultured bovine aortic endothelial cell line and primary human aortic endothelial cells to inhibit eNOS activation and up-regulate the expression of ICAM-1 and VCAM-1. These effects potentially could contribute to arterial lesion development and warrants further evaluations.

It should be pointed out that while diet-induced hyperlipidemia is an established risk factor for atherosclerosis, the effect of D76A on plasma lipid profile in our animals was relatively moderate, which alone may not explain the marked reduction in aortic lesions. Other mechanisms can be involved. For instance, liver secretion of D76A may help to sequester circulating myostatin in its inactive form; reducing its impact on endothelial production of adhesive molecules. In addition to regulation of liver lipogenesis, myostatin blockade by D76A may also change some of the properties of liver secretion, such as the size, composition, and redox state of the VLDL particles, which can result in less pro-atherogenic lipid particles in circulation. Furthermore, excessive lipid accumulation is often associated with tissue inflammation. Although D76A did not change the expression of selected common inflammatory markers in the liver (Figure S5) and in circulation (data not shown), we show that D76A reduced liver expression of TGFβ1 and apoCIII, both can be pro-atherogenic [49], [50]. D76A also reduced liver expression of stress marker CHOP-10 and HO-1, implying less oxidative damage in the liver which can alter the antioxidant defense in secreted lipid particles as well as other systemic redox regulators. Further studies are required to investigate these and other possible mechanisms to fully understand the remarkable phenotype observed in this study.

The pioneering work of Lee and others have emphasized the important evolutionary role of myostatin as a chalone that restrains unbridled muscle growth [31]. The findings of the current study suggest that myostatin may also promote hepatic fat stores and facilitate vessel wall wound healing; both can be important pro-survival evolutionary adaptations during periods of nutrient paucity. However, in the contemporary period of nutrient excess coupled with physical inactivity, the same function of myostatin could contribute to metabolic dys-regulations [51]–[55].

In summary, this work has demonstrated that (*i*) myostatin may participate in signal regulation and modulate metabolic outcomes through direct interactions with target cells in the liver and the aorta; (*ii*) myostatin antagonists might have a therapeutic role in the prevention or treatment of hepatosteatosis and atherosclerosis. These findings set the stage for further investigations of the potential therapeutic applications of myostatin antagonists for metabolic disorders as well as the mechanisms by which myostatin signaling pathways intersect with the metabolic regulatory pathways.

## Supporting Information

Figure S1 Expression of myostatin propeptide (nt 222–214, NM_010834) in the liver (left panel) and quadriceps muscle (right panel) between vehicle and D76A-treated animals, normalized to the expression of house-keeping gene HPRT. Each bar represents one individual animal.
(TIF)

Figure S2 Effects of myostatin inhibition by AAV-D76A on body composition. A: Time-dependent changes in body lean mass after injection of AAV-D76A during the first 7 weeks while the animals were fed normal chow. B: The net increase in total lean (left) and fat (right) mass during the first seven weeks (left panel), the 12 week of high fat diet (middle panel), and total experimental period (right panel). Results of A&B were obtained by NMR (mean +/− SE, N = 10, *p<0.05). C: Tissue weight expressed as percentage of total body mass (means +/− se, n = 10). Mean body mass was not significantly different between the two groups (43.29+/−2.96 g and 41.49+/−3.62 g, for vehicle- and D76A-treated groups, respectively, n = 10).
(TIF)

Figure S3 Effects of AAV-D76A on aorta lesion accumulation: additional *en face* aortic images supplemental to Figure 1. The microphotographs were taken on a different facility and more yellowish background color was recorded. Lipid-rich lesions are stained red with Sudan IV. The results clearly illustrated a reduction of aortic lesions in D76A-treated mice compared to the vehicle-treated ones.
(TIF)

Figure S4 Trichrome staining of lesions from the vehicle and D76A- treated mice. Red stains for muscle, including the smooth muscle in the fibrous cap at the lumen side, blue stains for collagen, white foamy structure indicates lipid-rich foam cell deposits, needle-like structures are cholesterol clefts from necrotic lipid core. Results are representative of five animals of each group.
(TIF)

Figure S5 Changes in liver transcripts after myostatin inhibition by AAV-D76A. Liver mRNA was analyzed by RT-qPCR for expression of genes downstream of TGFb/mystatin signaling: TGFβ1, ApoCIII, ANG-II and PAI-I (lower panel), stress-related genes: BIP, CHOP, and HO-1 (upper panel), and inflammatory genes: MCP-1, TNFα, and CRP (middle panel). Results are shown as means +/− se, n = 10, *t* test).
(TIF)

Figure S6 Primary mouse hepatocytes were isolated by the portal vein collagenase perfusion method as described (Li WC, Ralphs KL, Tosh D. Isolation and culture of adult mouse hepatocytes. Methods Mol Biol. 2010;633∶185–96. PMID: 20204628). After attachment, medium was replaced by serum-free M199 overnight. Myostatin (mstn) was added the next morning and incubated for 5 hours before RNA harvest. Expression of ActRIIB (NM_007397) was measured by qPCR (f-primer: CATTGCTGCCGAGAAACGAG; r-primer: TCCACGTGATGATGTTCCCC ). Exogenous myostatin induces expression of its own receptor ActRIIB in a bell-shape like dose-dependent manner.
(TIF)
