# Supplementary figures and images for: AAV-Mediated Administration of Myostatin Pro-Peptide Mutant in Adult Ldlr Null Mice Reduces Diet-Induced Hepatosteatosis and Arteriosclerosis
Source: PLoS One. 2013 Aug 1;8(8):e71017. doi: 10.1371/journal.pone.0071017 (PMC3731267; doi:10.1371/journal.pone.0071017)

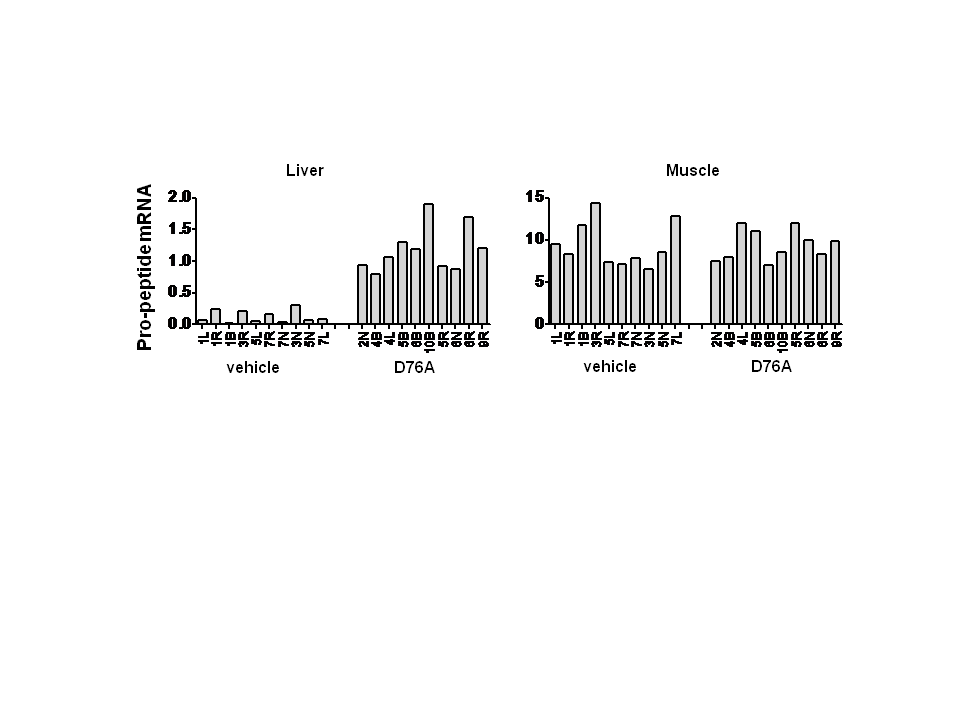

Supplement: Figure S1 — Expression of myostatin propeptide (nt 222–214, NM_010834) in the liver (left panel) and quadriceps muscle (right panel) between vehicle and D76A-treated animals, normalized to the expression of house-keeping gene HPRT. Each bar represents one individual animal. (TIF) [file pone.0071017.s001.tif]

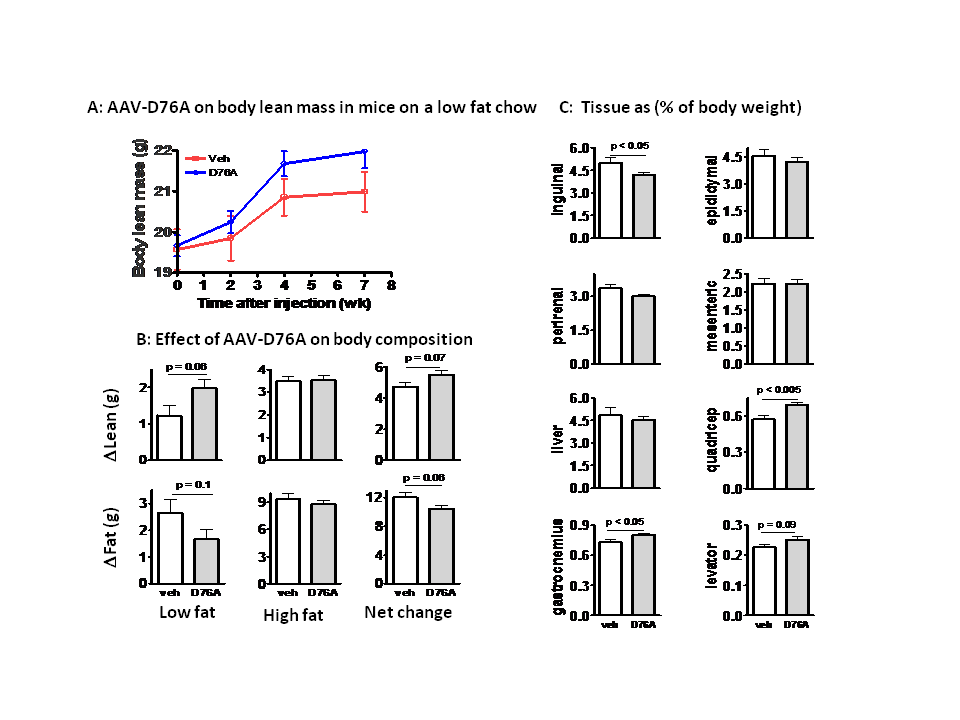

Supplement: Figure S2 — Effects of myostatin inhibition by AAV-D76A on body composition. A: Time-dependent changes in body lean mass after injection of AAV-D76A during the first 7 weeks while the animals were fed normal chow. B: The net increase in total lean (left) and fat (right) mass during the first seven weeks (left panel), the 12 week of high fat diet (middle panel), and total experimental period (right panel). Results of A&B were obtained by NMR (mean +/− SE, N = 10, *p<0.05). C: Tissue weight expressed as percentage of total body mass (means +/− se, n = 10). Mean body mass was not significantly different between the two groups (43.29+/−2.96 g and 41.49+/−3.62 g, for vehicle- and D76A-treated groups, respectively, n = 10). (TIF) [file pone.0071017.s002.tif]

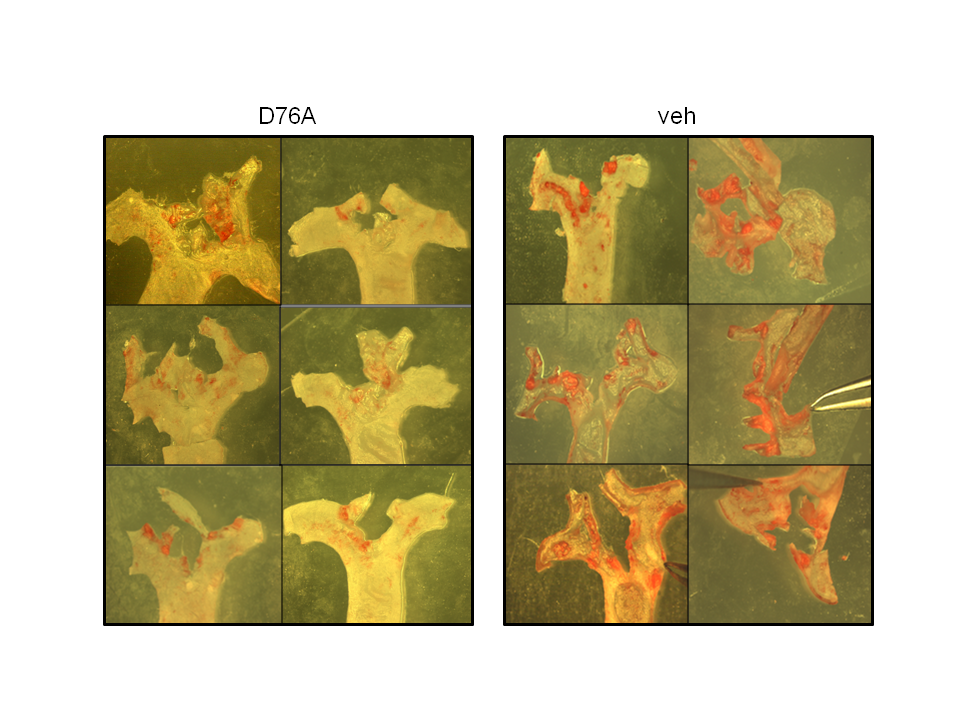

Supplement: Figure S3 — Effects of AAV-D76A on aorta lesion accumulation: additional en face aortic images supplemental to Figure 1. The microphotographs were taken on a different facility and more yellowish background color was recorded. Lipid-rich lesions are stained red with Sudan IV. The results clearly illustrated a reduction of aortic lesions in D76A-treated mice compared to the vehicle-treated ones. (TIF) [file pone.0071017.s003.tif]

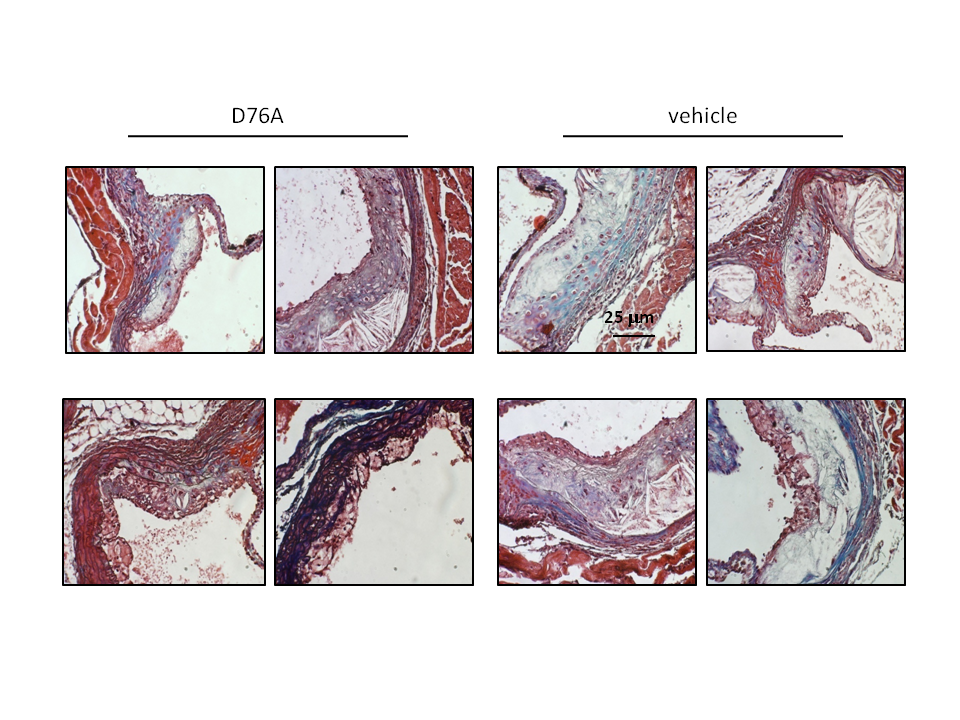

Supplement: Figure S4 — Trichrome staining of lesions from the vehicle and D76A- treated mice. Red stains for muscle, including the smooth muscle in the fibrous cap at the lumen side, blue stains for collagen, white foamy structure indicates lipid-rich foam cell deposits, needle-like structures are cholesterol clefts from necrotic lipid core. Results are representative of five animals of each group. (TIF) [file pone.0071017.s004.tif]

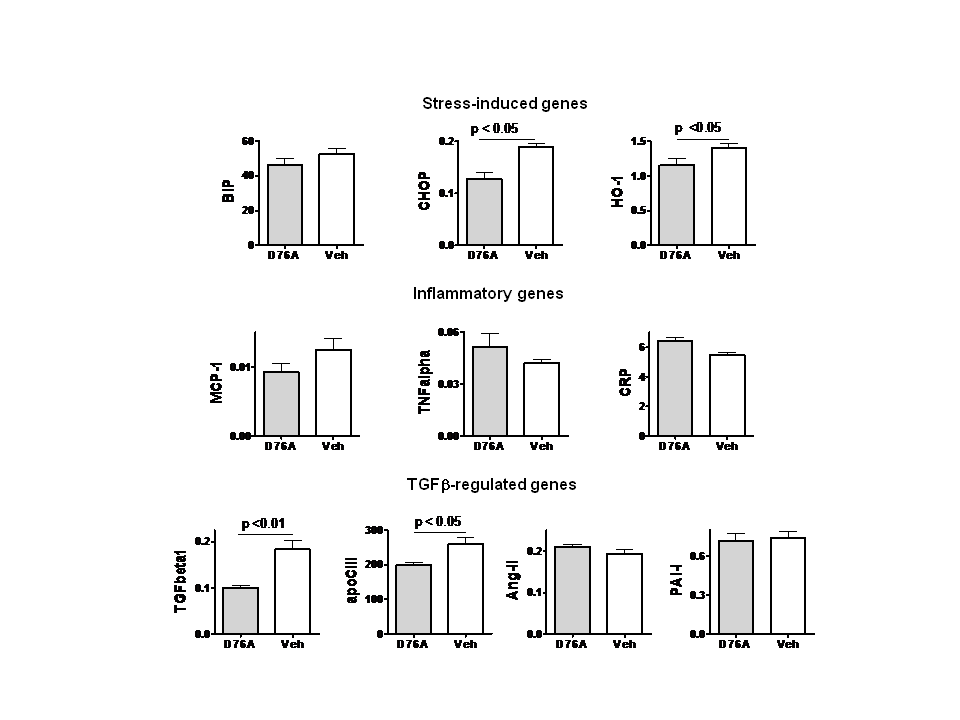

Supplement: Figure S5 — Changes in liver transcripts after myostatin inhibition by AAV-D76A. Liver mRNA was analyzed by RT-qPCR for expression of genes downstream of TGFb/mystatin signaling: TGFβ1, ApoCIII, ANG-II and PAI-I (lower panel), stress-related genes: BIP, CHOP, and HO-1 (upper panel), and inflammatory genes: MCP-1, TNFα, and CRP (middle panel). Results are shown as means +/− se, n = 10, t test). (TIF) [file pone.0071017.s005.tif]

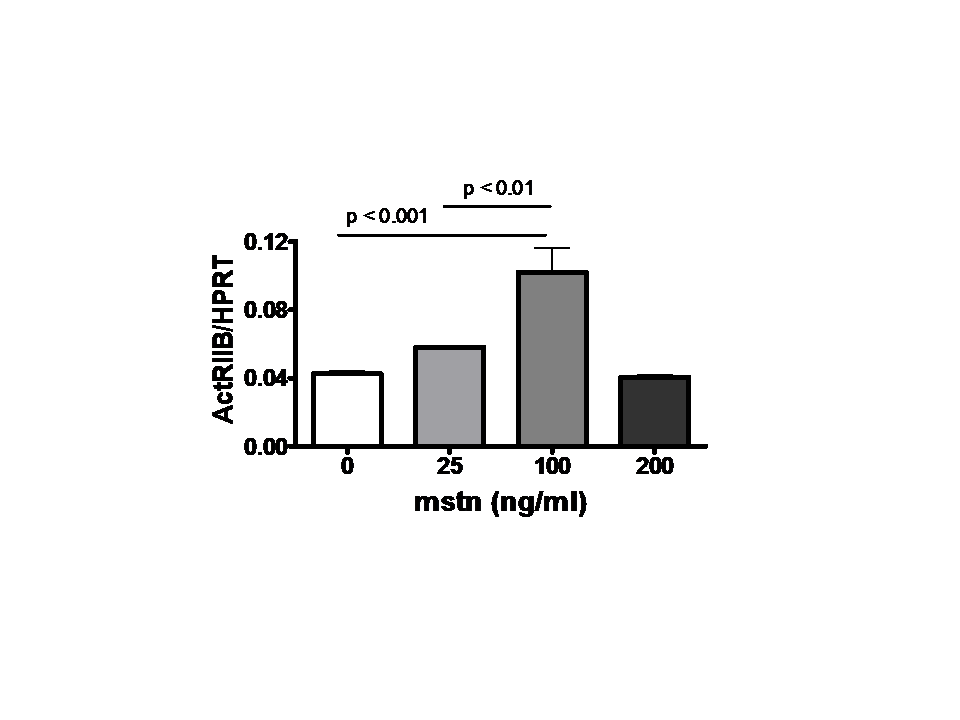

Supplement: Figure S6 — Primary mouse hepatocytes were isolated by the portal vein collagenase perfusion method as described (Li WC, Ralphs KL, Tosh D. Isolation and culture of adult mouse hepatocytes. Methods Mol Biol. 2010;633∶185–96. PMID: 20204628). After attachment, medium was replaced by serum-free M199 overnight. Myostatin (mstn) was added the next morning and incubated for 5 hours before RNA harvest. Expression of ActRIIB (NM_007397) was measured by qPCR (f-primer: CATTGCTGCCGAGAAACGAG; r-primer: TCCACGTGATGATGTTCCCC ). Exogenous myostatin induces expression of its own receptor ActRIIB in a bell-shape like dose-dependent manner. (TIF) [file pone.0071017.s006.tif]
